# Supplementary material for: Genotyping cognate Plasmodium falciparum in humans and mosquitoes to estimate onward transmission of asymptomatic infections
Source: Nat Commun. 2021 Feb 10;12:909. doi: 10.1038/s41467-021-21269-2 (PMC7875998; doi:10.1038/s41467-021-21269-2)
Supplement: Supplementary file 1 — Supplementary Information [file 41467_2021_21269_MOESM1_ESM.pdf]

Supplementary Methods

Additional sample processing methodology

Participant dried blood spot (DBS) samples and mosquito abdomens were shipped to Duke University in Durham, North Carolina, where they were processed to determine *P. falciparum* infection status and haplotypes. Mosquito parts were individually ground in 1% Saponin using a micro tube homogenizer system fitted with a pestle, and the homogenate was transferred to unique wells of a deep 96-well plate. Single 6mm punches from the DBS were likewise distributed in deep well plates and genomic DNA (gDNA) was extracted from mosquito and DBS samples using a Chelex-100 protocol.[1] As described in Taylor *et al.,*[2] each sample was tested in duplicate for *P. falciparum* parasites using a duplex TaqMan real-time PCR (qPCR) assay targeting the *P. falciparum* pfr364 motif and the human β-tubulin gene.

P. falciparum positive DBS gDNA was prepared for genotyping based on qPCR Ct-values. Samples with Ct 25 - 30 were applied to Genomic DNA Clean & Concentrator-10 columns, and for samples with Ct >30, gDNA from a second punch of each identical DBS was added to the initial sample and the total applied to RNA Clean & Concentrator-5 columns. *P. falciparum* positive mosquito gDNA samples were applied to DNeasy PowerClean Pro Cleanup columns and the eluate concentrated by EtOH precipitation.

Library preparation for sequencing followed methods described in Nelson *et al.*[3] but with the following exceptions. PCR1 reactions contained 300 nM of each primer and 2 μL of template gDNA when DBS sample Ct was < 25, 5 μL when Ct 25 - 30, 9 μL when Ct > 30, and 7 μL for mosquito gDNA. PCR2 reactions contained 2 μL template when DBS sample Ct < 25, 9 μL when Ct ≥ 25, and 3 μL for mosquito template. Dual-indexed libraries were prepared for the polymorphic *P. falciparum* parasite gene targets encoding apical membrane antigen-1 (*pfama1*) and circumsporozoite protein (*pfcsp*), then pooled and sequenced on an Illumina MiSeq platform.[4] The specific primers used for sequencing are provided (Tables S7 and S8).

Additional haplotype calling information for samples for *pfama1* and *pfcsp*

pfama1 and *pfcsp* haplotypes were called using the amplicon deep sequencing reads. As in Nelson *et al.*,[3] CutAdapt, Trimmomatic, and BBmap were used to trim *pfama1* and *pfcsp* primers and adapters, quality filter reads with an average Phred Quality Score < 15 over a sliding window of 4 nucleotides, remove reads less than 80 nucleotides long, and map sample reads to the 3D7 reference sequences for *pfama1* and *pfcsp* to differentiate between the two gene targets.[3,5–7] Quality-filtered reads were input into the R (version 3.6.1) package DADA2 (version 1.8) to join paired-end reads, perform an additional quality filter based on modeled error frequency, call haplotypes, and remove chimeras.[8,9] This process outputted haplotypes (distinct sequences of the *pfama1* or *pfcsp* gene target) to be used as a measure of parasite genetic diversity. Because sequencing low parasite densities has been associated with an increased risk of haplotype false discovery,[10] haplotypes were further filtered in order to mitigate the risk of false discovery by removing haplotypes from a sample that met any of the following criteria: (i) supported by < 250 reads within the sample; (ii) supported by < 3% of the sample's total read depth; (iii) deviation from the expected nucleotide length of 300 for *pfama1* or 288 for *pfcsp*; or (iv) a minority haplotype distinguished by a one single-nucleotide polymorphism (SNP) difference from another haplotype within the sample that had a read depth > 8 times the read depth of the minority haplotype.[10] Finally, we removed haplotypes from the overall population if each haplotype was defined by a single variant position that was only variable within that haplotype.

We defined censoring criteria empirically by analyzing sequences of *pfama1* and *pfcsp* obtained from controlled mixtures of *P. falciparum* strains 3D7, V1/S, 7g8, Dd2, and FCR3. The figure and table show results for the *pfcsp* region sequenced (S1 Fig). *P. falciparum* V1/S and Dd2 strains were identical within the *pfcsp* region sequenced, so results are presented with the

reads combined. To develop haplotype censoring criteria, the controls were sequenced in differing proportions (control mixtures C1-C6). After quality-filtering reads and applying the haplotype censoring criteria, the final percentage of reads of each strain was similar to what was expected from the control mixtures, as indicated in the figure and table. Because the censoring criteria filtered out reads that were present in < 3% of the sample's total reads, the 3D7 and 7g8 controls were filtered out in control mixture 6. Similar results were produced for *pfama1*.

Supplementary Results

Comparison of target variant positions with prior studies

Across all samples, we compared the variant positions that we identified in the sequenced fragments of *pfcsp* and *pfama1* with those identified in prior studies. To do so, we compiled variant positions in these fragments from PlasmoDB (accessed August 1, 2019),[11] the Pf3k database (accessed July 30, 2019),[12] as well as an external data set (Neafsey *et al.).*[13] For the latter, we downloaded raw sequencing reads and processed these with the haplotype inference criteria described above (S2 Fig).[13] Through these searches, the number of variant positions in our sequenced fragment of *pfcsp* was 30 in PlasmoDB, 44 in Pf3k, and 39 in Neafsey *et al.*[11–13] Overall, these databases yielded a total of 57 variant positions, and 37 of these were among the 72 nucleotide positions that we identified in our sequences.

Haplotype distributions between sample types

Because low parasite density samples were sequenced and strict filtering criteria were used, some samples failed sequencing and were not genotyped for *pfcsp* or *pfama1*. A total of 1242 samples were sequenced across 902 asymptomatic participant infections, 137 symptomatic participant infections, and 203 mosquito abdomens. After censoring criteria was applied, we identified *pfcsp* haplotypes in 185 mosquito abdomens, 733 asymptomatic

infections, and 128 symptomatic infections (S3 Fig). For *pfama1,* we identified haplotypes in 177 mosquito abdomens, 611 asymptomatic participants, and 113 symptomatic participants. Based on these numbers, *pfcsp* had a sequencing failure rate of 196/1242 (15.78%) and *pfama1* had a sequencing failure rate of 341/1242 (27.46%). Using the Wilcoxon Rank Sum test with continuity correction, there was a statistically significant correlation between parasite density and sequencing failure for *pfcsp* (W=151332, *p*-value < 0.001) and *pfama1* (W=200703, *p*-value < 0.001), with more sequencing failures for *pfama1* than *pfcsp*. While there were statistically significant differences between parasite density and the likelihood of sequencing failure, a directed acyclic graph (DAG) representing missingness due to sequencing failure indicated that restricting the data set to samples that passed sequencing was unlikely to produce missing data bias (S4 Fig); however, as a precaution to account for lower parasite density samples potentially being biased towards sequencing failure and a form of missing at random bias, we included a covariate in our models for parasite density.

Inferred *pfama1* haplotypes across samples

For *pfama1*, 348 unique haplotypes were identified across 177 mosquito abdomens, 611 asymptomatic participants, and 113 symptomatic participants. Haplotypes produced from *pfama1* had a median MOI of 7 for mosquito abdomens, 1 for symptomatically-infected participants, and 2 for asymptomatically-infected participants.

Functional form assessment for continuous variables

A functional form assessment was conducted for continuous variables included in the models: parasite density in the participant samples, participant age at study enrollment, and mosquito abundance. The functional form assessment indicated that the optimal coding for parasite density was linear and rescaled to have a mean value of 0.0 due to its interpretability

and similar functional form (Table S3). For participant age, the categorical coding (categorized: <5 years, 5-15 years, >15 years) was the best choice, because it had the lowest Akaike information criteria (AIC) value, fit the functional form, and was a commonly used coding of age in malaria literature (Table S4). For mosquito abundance, a binary coding was chosen (expressed as the total number of female *Anopheles* mosquitoes collected within the week following the participant infection stratified at <75 mosquitoes or ≥75 mosquitoes), because that functional form had the lowest AIC, was easily interpretable, and had a similar functional form to the variable (Table S5).

Within-participant modeling of transmissibility for *pfama1*

Using the *pfama1* haplotypes shared as a proxy for transmission, we selected 56 participants who suffered at least one asymptomatic and one symptomatic infection that passed genotyping for *pfama1*. The participants had multiple infections matched with mosquitoes consisting of 1197 participant-mosquito pairs. Mosquitoes were collected between 7 days before and 14 days after the participant infection and were within 3 kilometers of the participant's household. Asymptomatic infections (Median: 0.34) had a higher median proportion of pairings that shared at least one *pfama1* haplotype with a mosquito compared to symptomatic infections (Median: 0.25) across the participants (S13 Fig). In a multi-level logistic regression model controlling for parasite density and mosquito abundance, compared to symptomatic infections, asymptomatic infections had higher odds of sharing parasite haplotypes with infected mosquitoes [Odds Ratio (OR): 1.30, 95% Confidence Interval (CI): 0.63 to 2.69] (S14 Fig).

Probabilistic modeling of transmission across all participants for *pfama1*

For a more comprehensive analysis of all participants, we conducted an additional analysis of transmissibility using a probabilistic modelling framework. After applying time and

distance constraints to participant-mosquito pairings, the final *pfama1* analysis data set consisted of 3160 observations of participant-mosquito pairs found across 178 participants, 172 mosquitoes, and 36 households. 2537 pairs had a participant with an asymptomatic infection and 623 pairs had a participant with a symptomatic infection. The overall probability of transmission outcome measure, $P(TE_{all})$, ranged from 0.00 to 0.99 with a median of 0.00. Using the continuous coding of $P(TE_{all})$ and controlling for confounding covariates: parasite density in participant samples in parasites/μL, participant age, mosquito abundance, and village, we found that over 14 months participants with asymptomatic infections had an odds of participant-to-mosquito malaria transmission that was 1.22 (95% CI: 0.82 to 1.82) times the odds of transmission for participants with symptomatic infections (S15 Fig).

Supplementary Figures

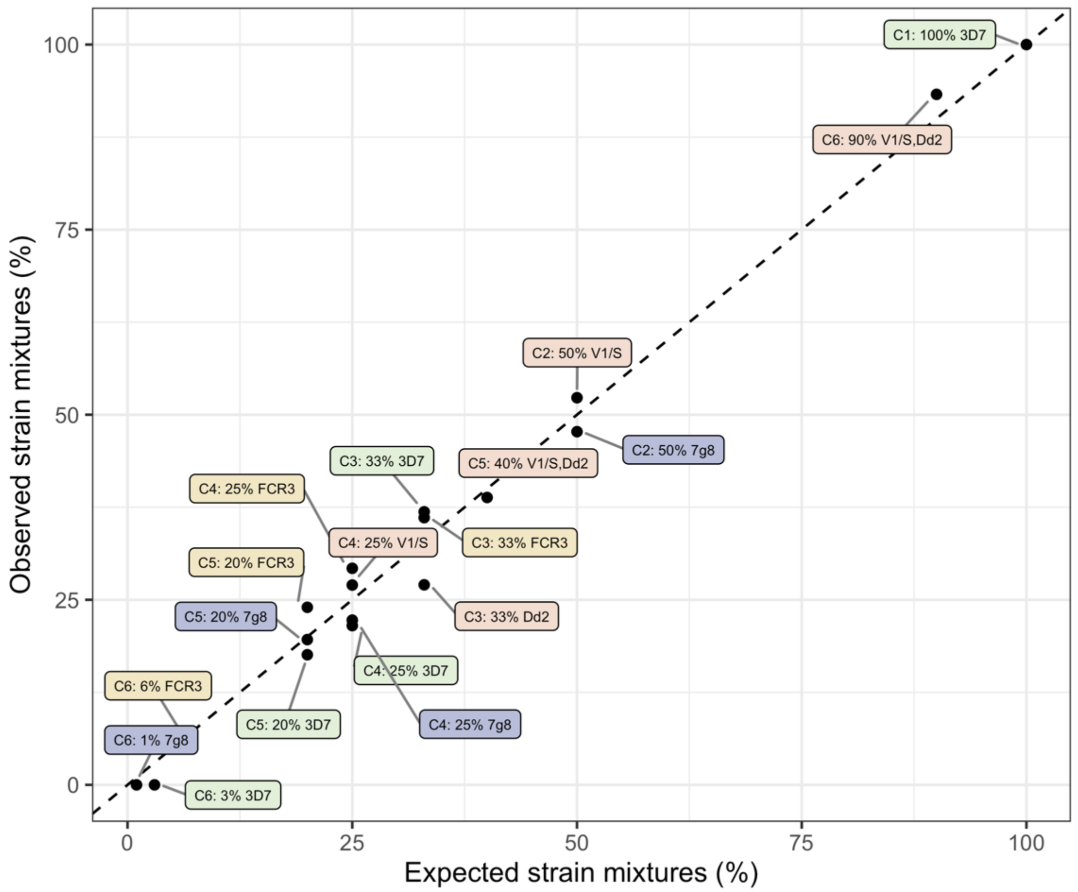

Control Mixture	Expected strain mixtures before sequencing (%)				Observed haplotype proportions after sequencing and haplotype censoring criteria applied Number of reads (%)			
	3D7	FCR3	V1/S or Dd2	7g8	3D7	FCR3	V1/S or Dd2	7g8
C1	100%	NA	NA	NA	21006 (100%)	0	0	0
C2	NA	NA	50%	50%	0	0	10444 (52%)	9528 (48%)
C3	33%	33%	33%	NA	5972 (37%)	5844 (36%)	4375 (27%)	0
C4	25%	25%	25%	25%	3590 (22%)	4882 (29%)	4505 (27%)	3714 (22%)
C5	20%	20%	40%	20%	3389 (18%)	4625 (24%)	7486 (39%)	3784 (20%)
C6	3%	6%	90%	1%	Censored	1886 (7%)	26155 (93%)	Censored

S1 Fig. Expected and observed *pfcsp* haplotype frequencies in control mixtures of

genomic DNA from *P. falciparum* reference lines. Expected strain mixtures were based on the input amounts of genomic DNA of each reference parasite strain. Strains V1/S and Dd2 share identical *pfcsp* haplotypes and therefore could not be resolved. Haplotypes in “C6” that mapped to 3D7 and 7g8 were censored because they were present in ≤ 3% in the overall read yield for that template. NA: not applicable.

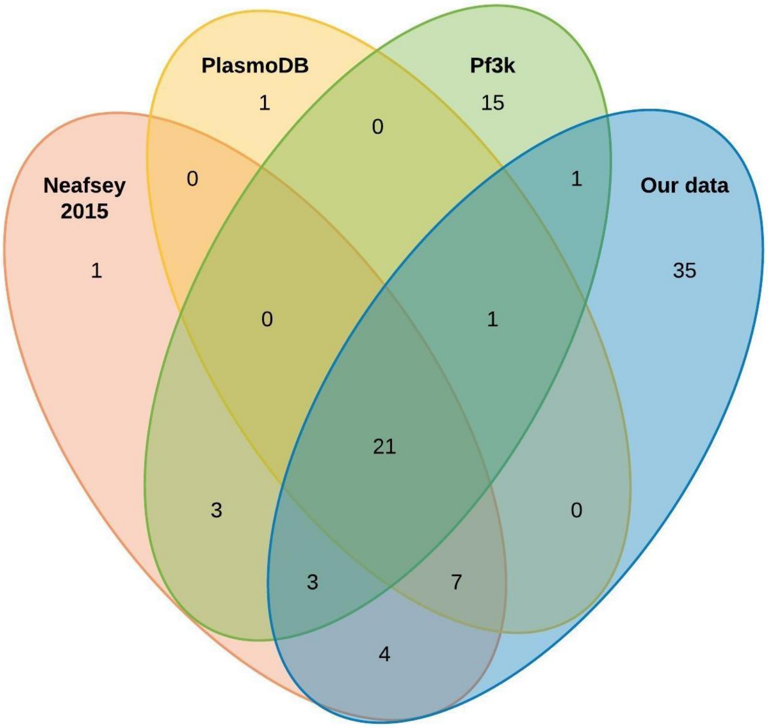

S2 Fig. Comparison of overlap in the variant nucleotide positions within the sequenced *pfcsp* fragment identified in our study and in prior studies. The total number of variant nucleotide positions for each set was: Neafsey *et al.* = 39, PlasmoDB = 30, Pf3k = 44, and this study = 72.[11–13]

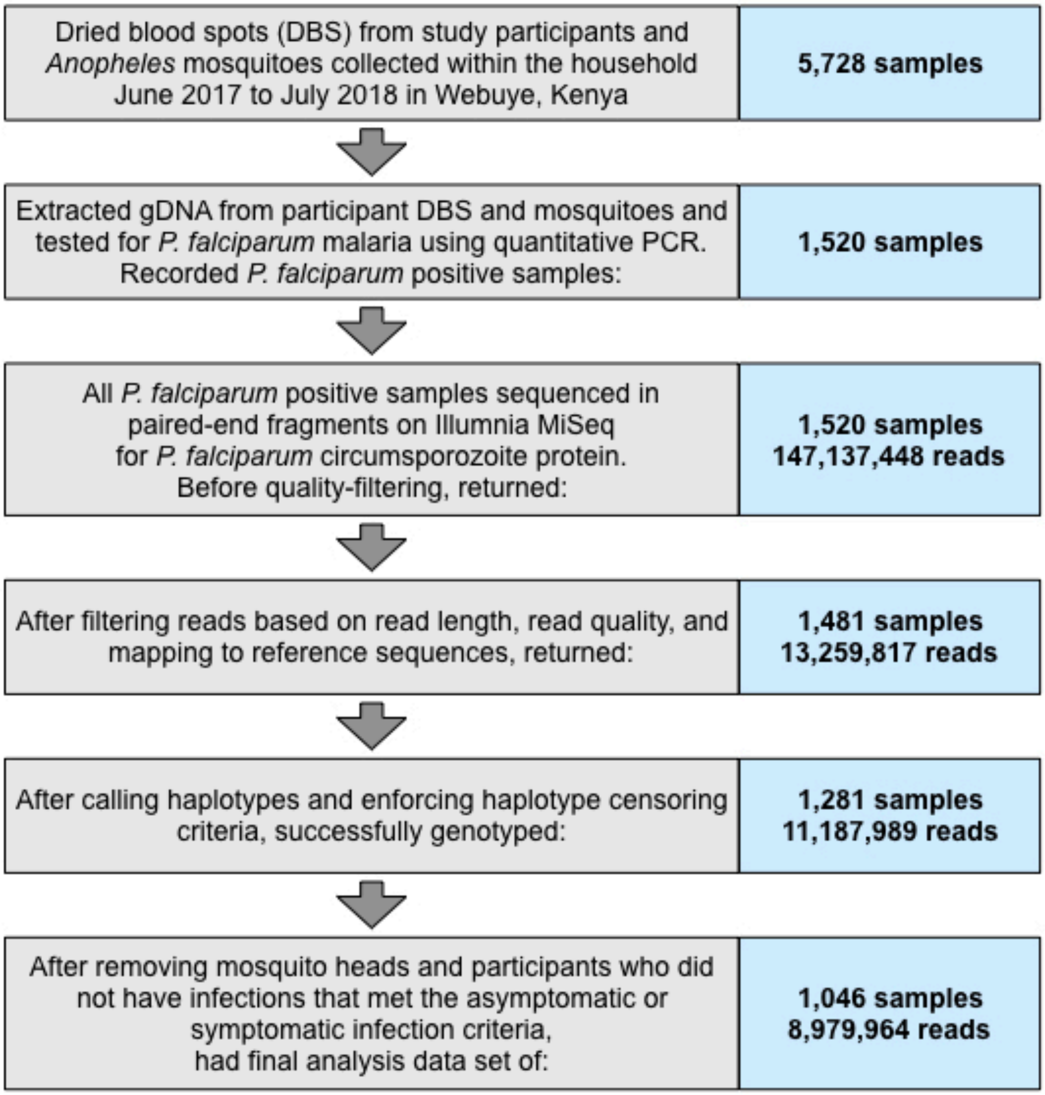

S3 Fig. Sample processing flow-diagram from original samples to censored, high-quality haplotypes. The number of samples and reads returned from each step of sample processing is shown for amplicon deep sequencing of *pfcsp*. The same process was done for *pfama1*.

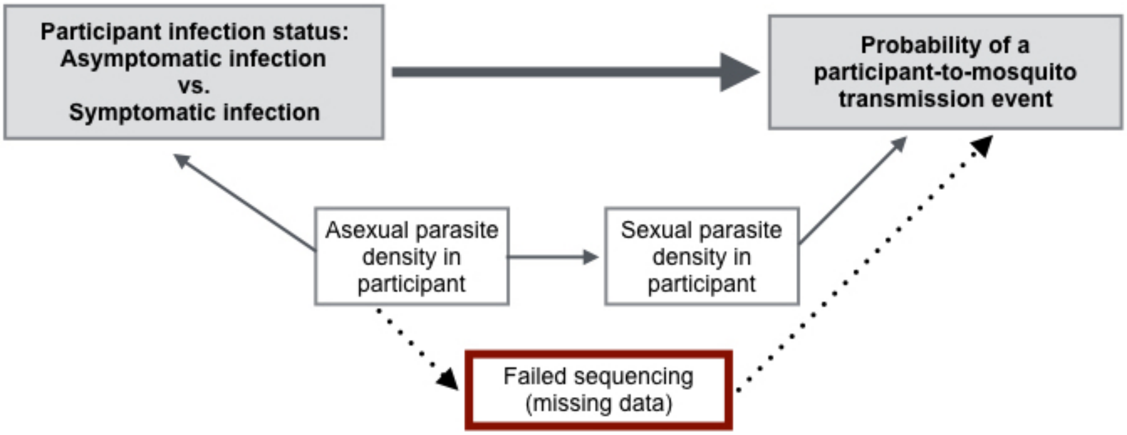

S4 Fig. Directed acyclic graph (DAG) investigating potential for missing data bias in samples that failed sequencing. A DAG was used to assess potential bias caused by data missing at random based on sequencing failure.

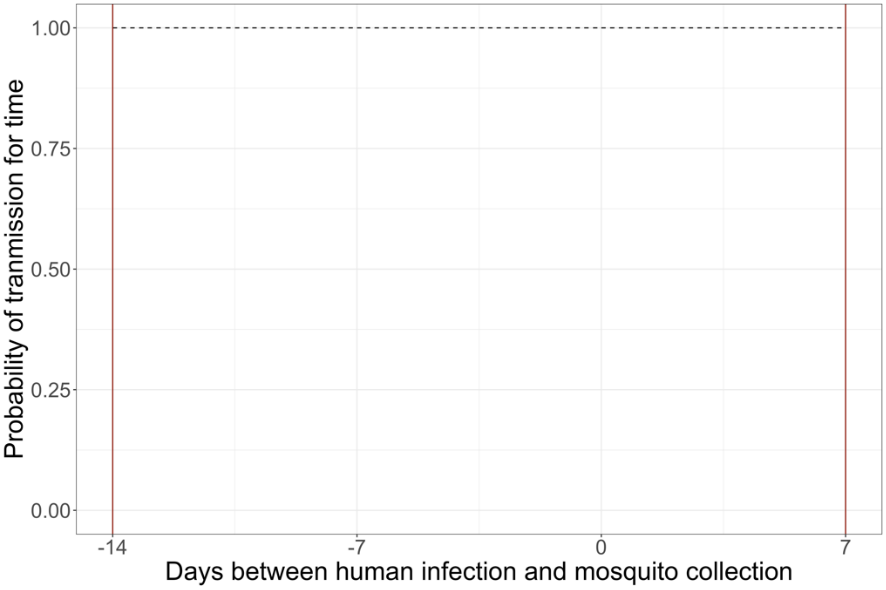

S5 Fig. Probability of transmission over time. The probability of transmission over time [$P(TE_t)$] distribution had a flat, high probability of transmission from -14 to 7 days to allow for each participant sample to have the same number of mosquito collections and the same probability of transmission within the time range. The distribution was restricted to only allow a transmission event to occur when a mosquito was collected within 14 days (i.e. -14 days) after the participant infection or 7 (i.e. +7 days) days prior to the participant infection. Any participant-mosquito pair within this time range, had $P(TE_t)$ = 1. Any time outside of the time range, had $P(TE_t)$ = 0.

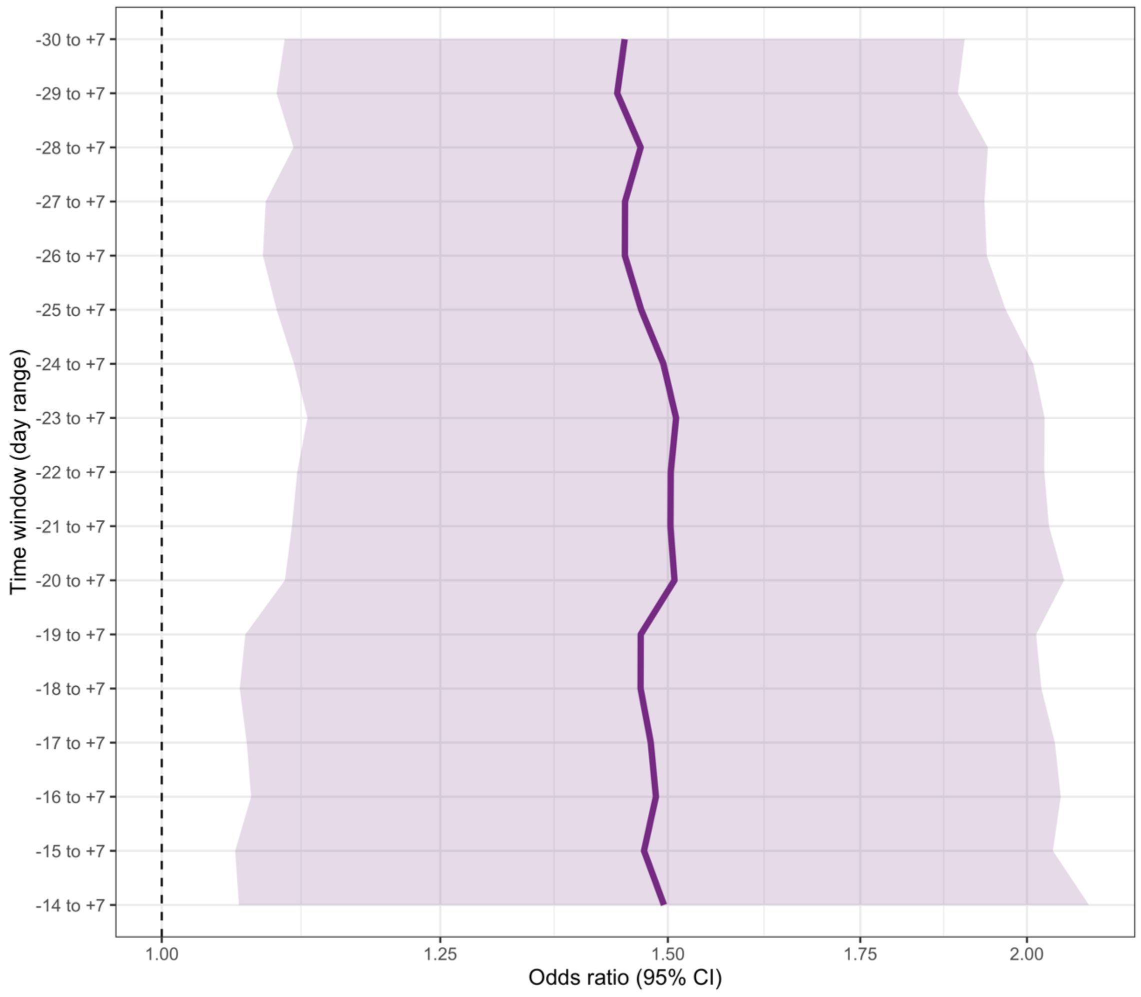

S6 Fig. Sensitivity analysis for probability of transmission over time. A sensitivity analysis was done to comparing different time windows for the probability of transmission over time [$P(TE_t)$] and the effect on the relationship observed. The distribution was restricted to only allow a transmission event to occur when a mosquito was collected within 30 to 14 days (i.e. -30 to -14 days) after the participant infection or 7 (i.e. +7 days) days prior to the participant infection. The multi-level logistic regression model was reran comparing the probability of transmission to mosquitoes across participants with asymptomatic compared to symptomatic infections using each time window (across all windows the total number of participant-mosquito pairings included ranged from N=3727 to 6133). Each time window is shown on the y-axis and the

associated odds ratio for transmission on the x-axis. The shaded area represents the 95% confidence interval around each odds ratio. The *pfcsp* haplotypes were used for this sensitivity analysis.

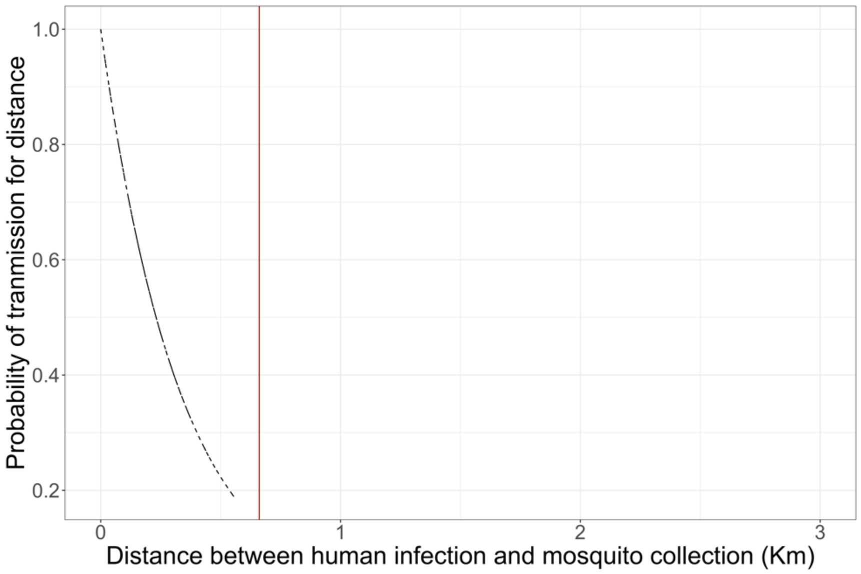

S7 Fig. Probability of transmission over distance. The distribution of the probability of transmission over distance [$P(TE_d)$] decreased to a low probability of transmission as the distance between the participant infection and mosquito collection increased. At any distance greater than 3 kilometers, estimated $P(TE_d)$ = 0, allowing transmission events to occur across households but not villages. The curve stops at 0.56 kilometers, because no participants and mosquitoes were collected within a village at a distance greater than 0.56 kilometers.

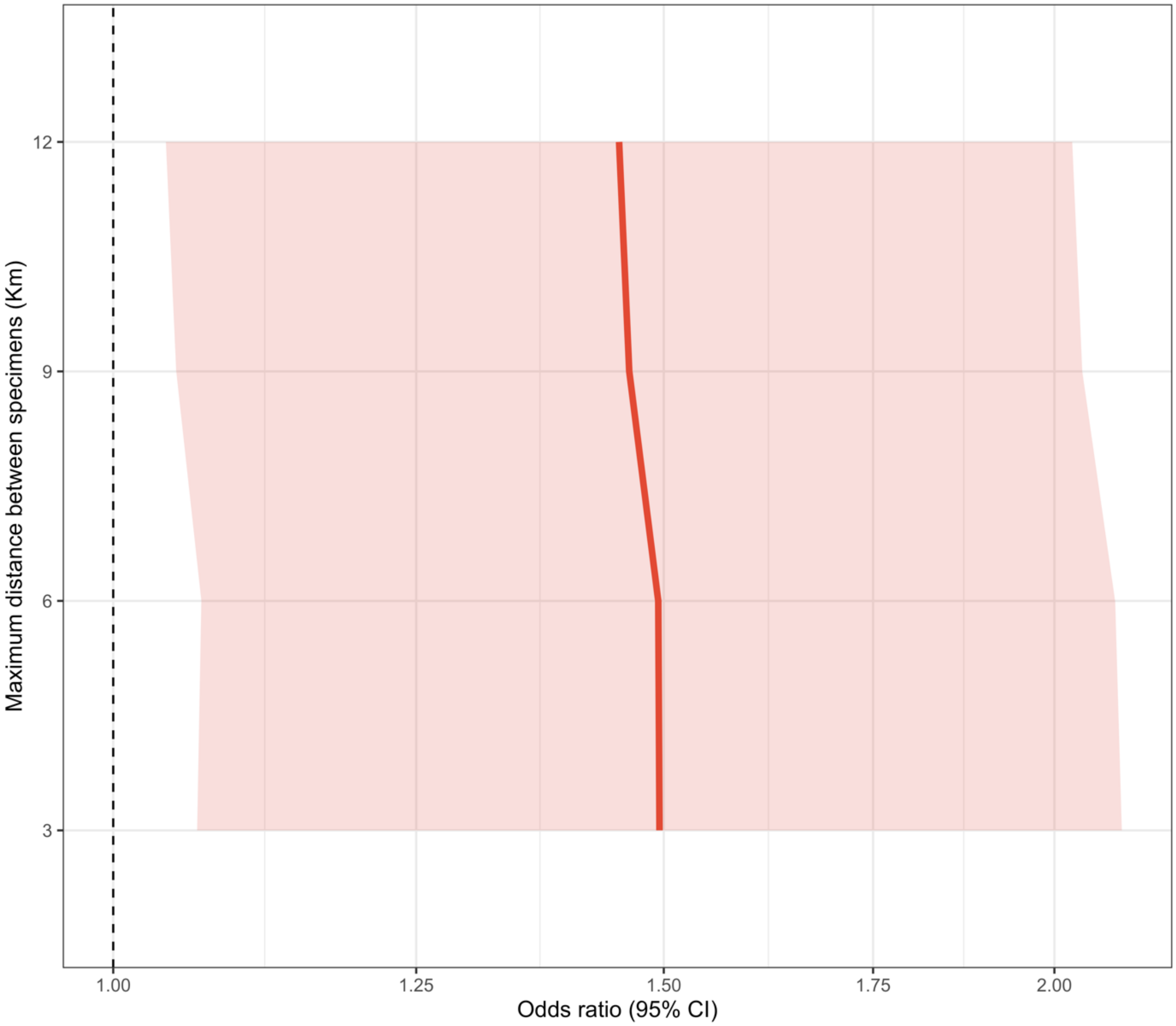

S8 Fig. Sensitivity analysis for probability of transmission over distance. A sensitivity analysis was done to comparing different distance cutoffs for the probability of transmission and the effect on the relationship observed. The multi-level logistic regression model was reran comparing the probability of transmission to mosquitoes across participants with asymptomatic compared to symptomatic infections using each distance cutoff (across all windows the total number of participant-mosquito pairings included ranged from N=3727 to 9911). Each maximum distance cutoff is shown on the y-axis and the associated odds ratio for transmission to mosquitoes on the x-axis. The shaded area represents the 95% confidence interval around

each odds ratio. The *pfcsp* haplotypes were used for this sensitivity analysis.

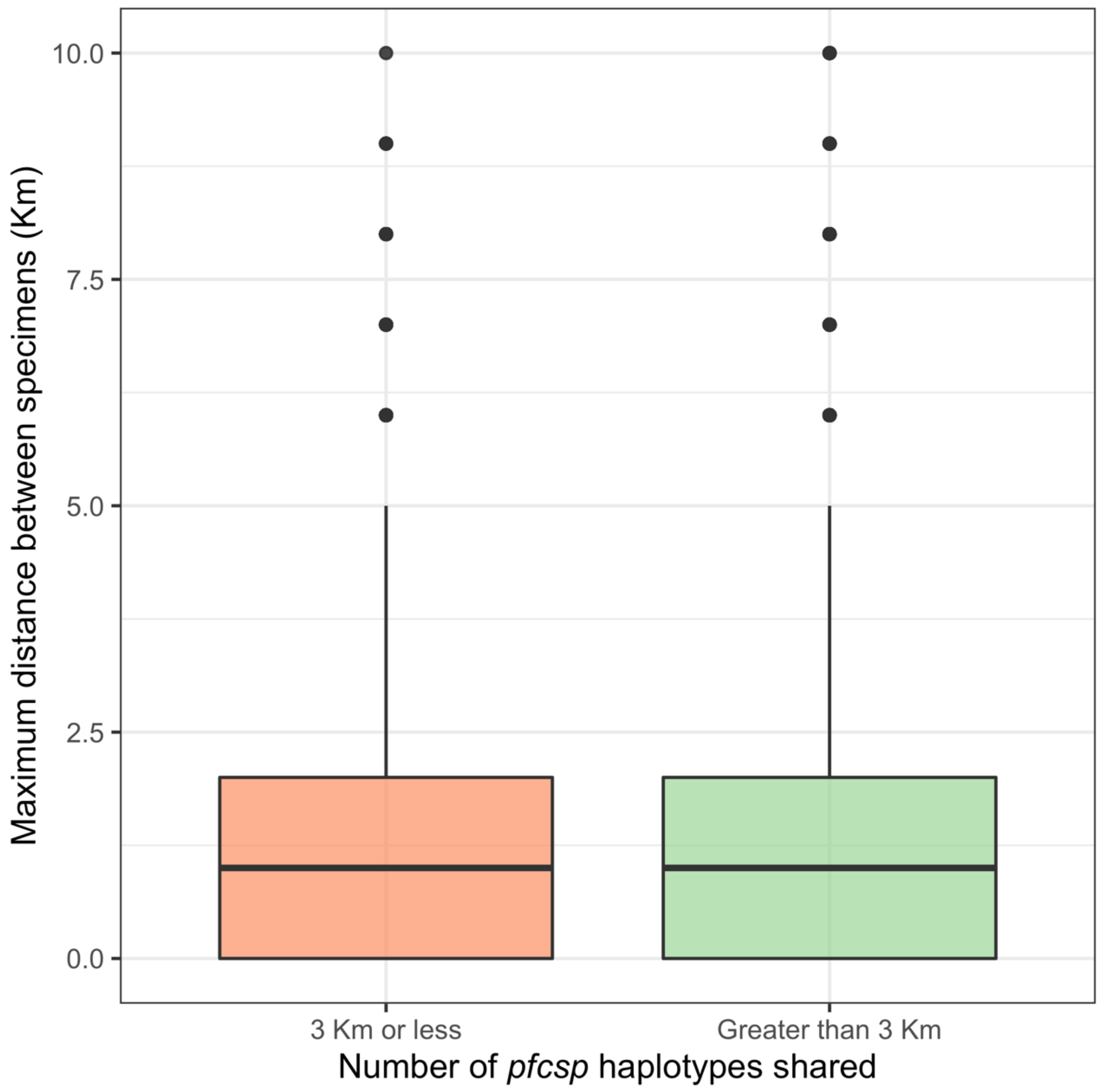

S9 Fig. Number of shared *pfcsp* haplotypes between participants and mosquitoes at < 3 kilometers and ≥ 3 kilometers. The number of pfcsp haplotypes shared between specimens collected at a distance < 3 kilometers (N=54,868 participant-mosquito pairings) and ≥ 3 kilometers (N=111,773 participant-mosquito pairings) was compared. The upper and lower sections of the box represent the 25th and 75th percentiles of the data respectfully. The bold center line indicates the median. Outliers are indicated by dots.

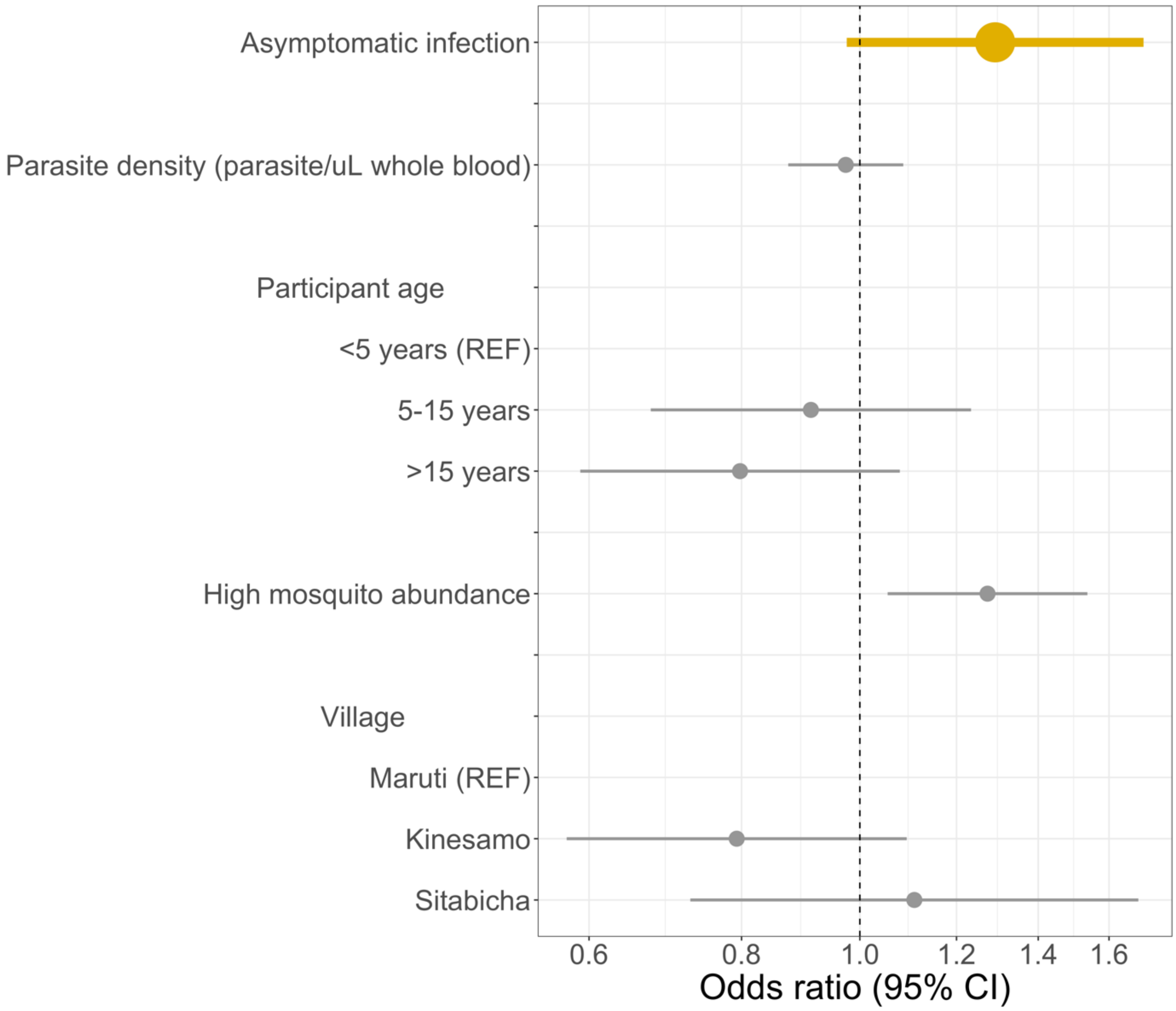

S10 Fig. Sensitivity analysis for probability of transmission over haplotypes. A sensitivity analysis was done using a different coding for the $P(TE_h)$ term where it was no longer calculated separately for *pfama1* and *pfcsp* but instead calculated as a combined value using both *pfama1* and *pfcsp* haplotypes. The multi-level logistic regression model was reran comparing the probability of transmission to mosquitoes across participants with asymptomatic compared to symptomatic infections (N=3970 participant-mosquito pairings). Data are presented as odds ratios (dots) with 95% confidence intervals (bars).

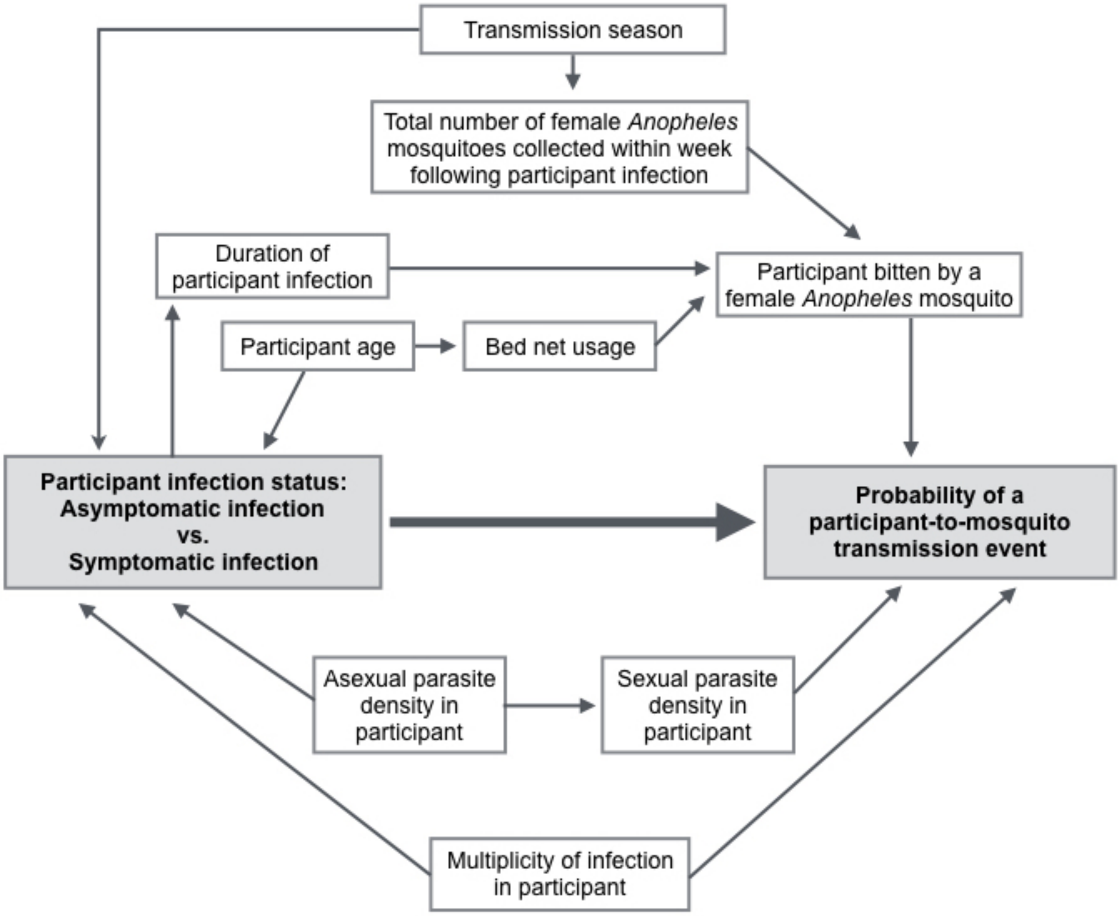

S11 Fig. DAG of causal relationship between a participant's symptomatic status and probability of a participant-to-mosquito transmission event. The DAG identified four confounding covariates that needed to be controlled for in assessing the effect of participants' malaria symptomatic status on the probability of a participant-to-mosquito transmission event: age, parasite density in the participant samples in parasites/μL, total number of female *Anopheles* mosquitoes collected within the week following the participant infection, and multiplicity of infection (MOI) in participants. MOI was controlled for in Equation 3 for $P(TE_h)$.

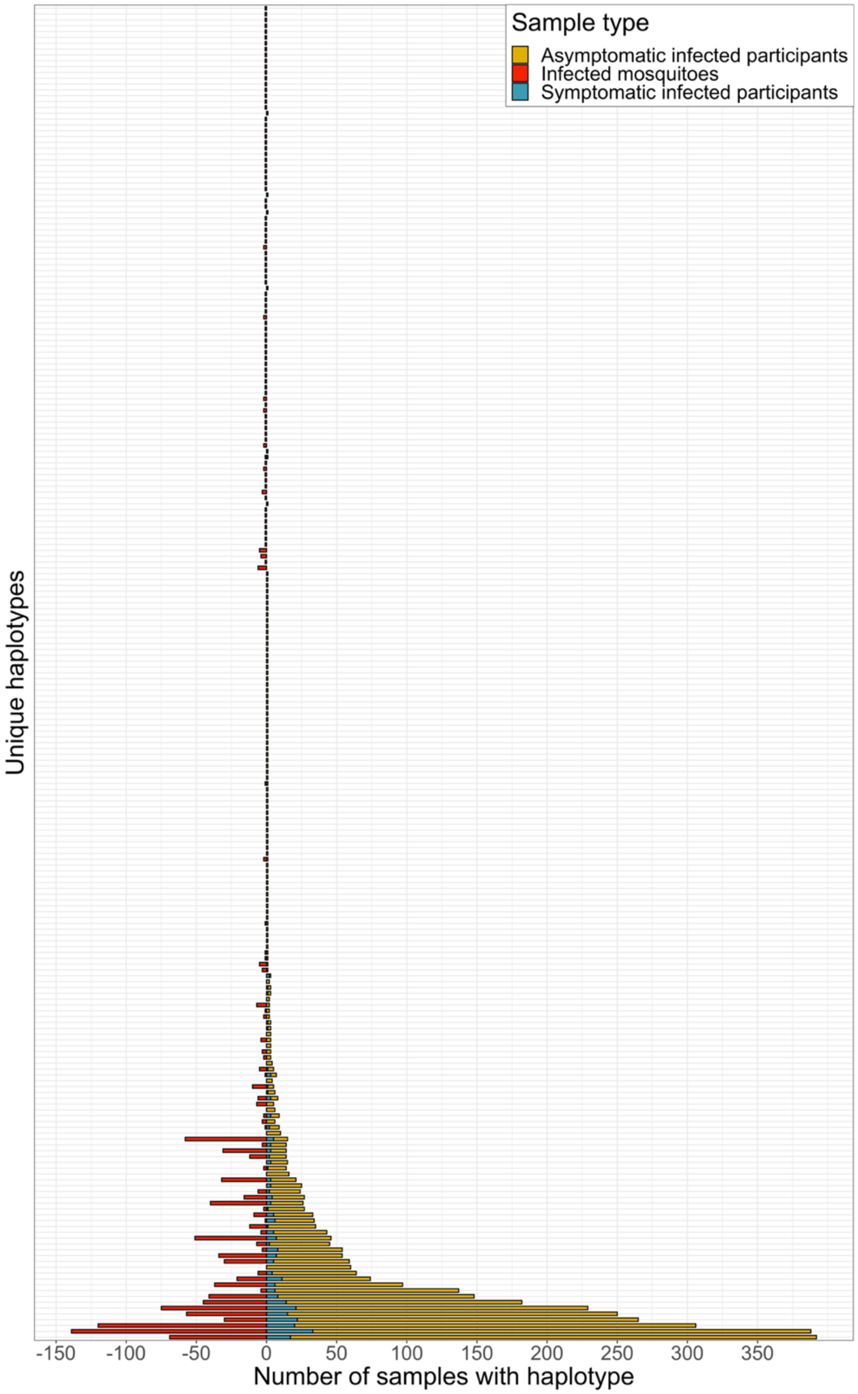

S12 Fig. Haplotype distribution across sample types for *pfcsp.* The full distribution of *pfcsp* haplotypes across mosquitoes, asymptomatic infections, and symptomatic infections is shown here. This plot shows all *pfcsp* haplotypes regardless of how many samples they were found in.

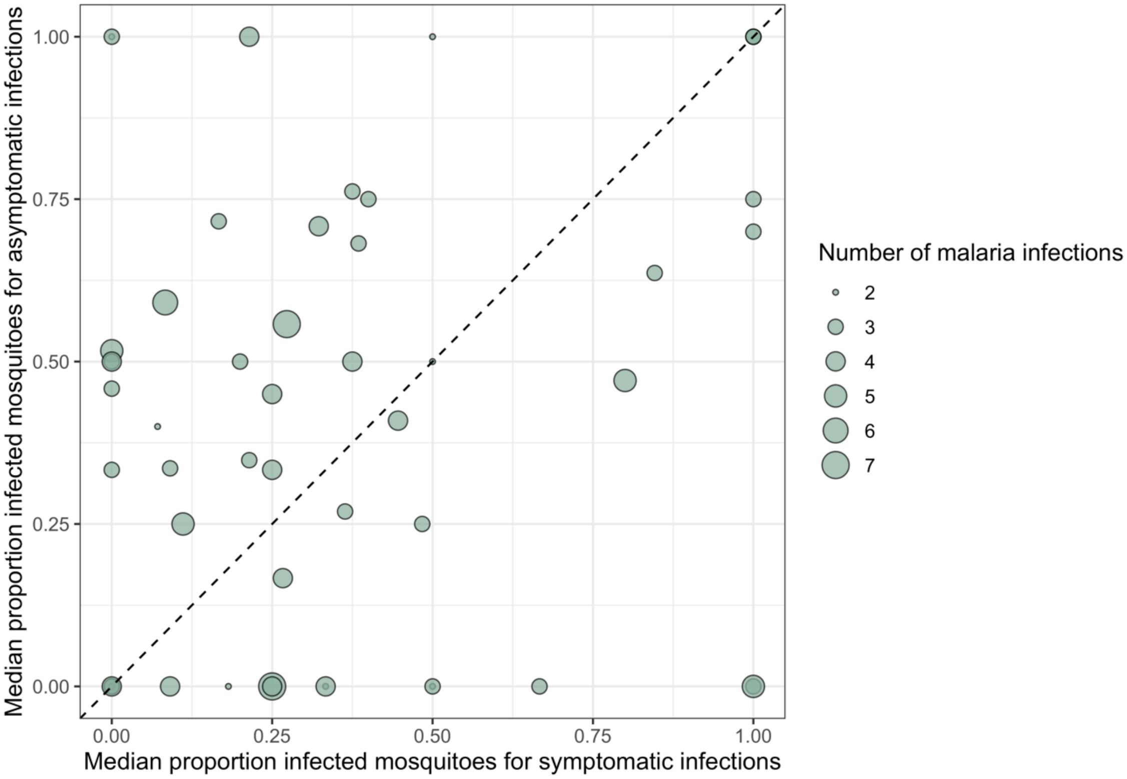

S13 Fig. Comparison of likelihood of transmission to mosquitoes for participants with both asymptomatic and symptomatic infections using the *pfama1* gene target. For each participant (N=56), the median proportion of pairings with a mosquito that shared a minimum of one haplotype was calculated for asymptomatic and symptomatic infections to represent the average likelihood of transmission to a mosquito. Using the *pfama1* gene target, asymptomatic infections had a higher median likelihood of transmission, as indicated by the higher number of dots to the left of the dotted diagonal line; however, this difference was not statistically significant as shown in S14 Fig.

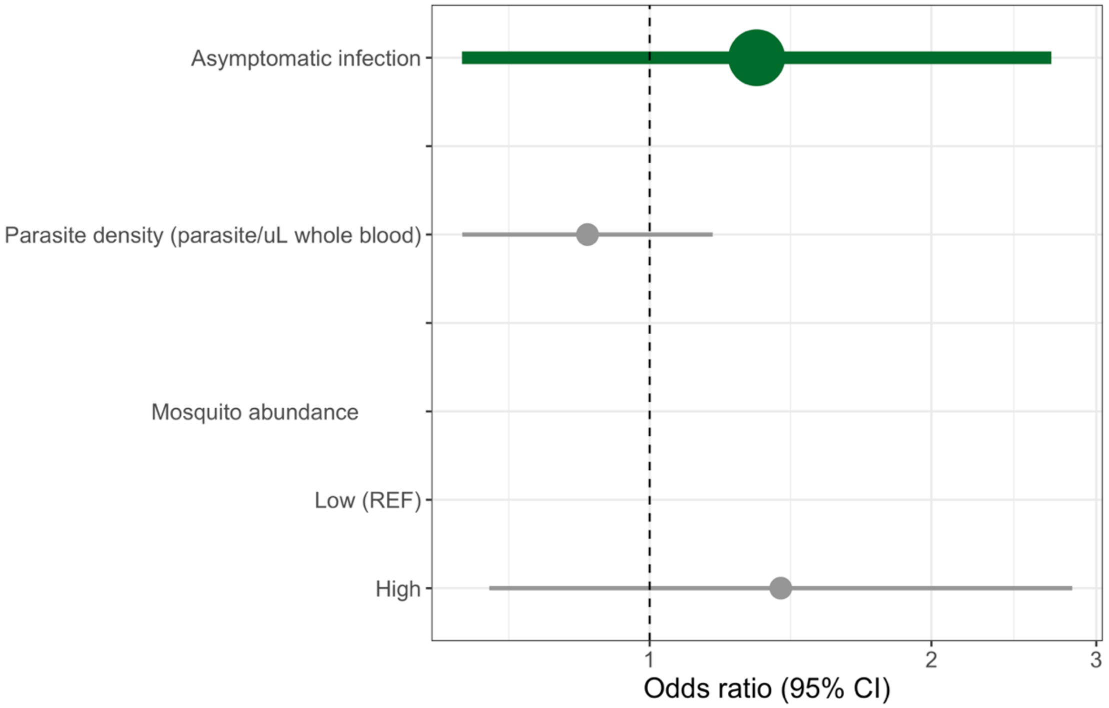

S14 Fig. Comparison of likelihood of transmission to mosquitoes for participants with both asymptomatic and symptomatic infections using the *pfama1* gene target. We ran a multi-level logistic regression using the continuous coding of the proportion of participant-mosquito pairings (N=1197) that shared at least one *pfama1* haplotype for each infection. The model controlled for covariates: parasite density in the participant samples in parasites/μL (linear) and the mosquito abundance (binary: <75 mosquitoes, ≥75 mosquitoes). Model results suggested higher odds of asymptomatic compared to symptomatic malaria transmission to mosquitoes, but results were not statistically significant. Data are presented as odds ratios (dots) with 95% confidence intervals (bars).

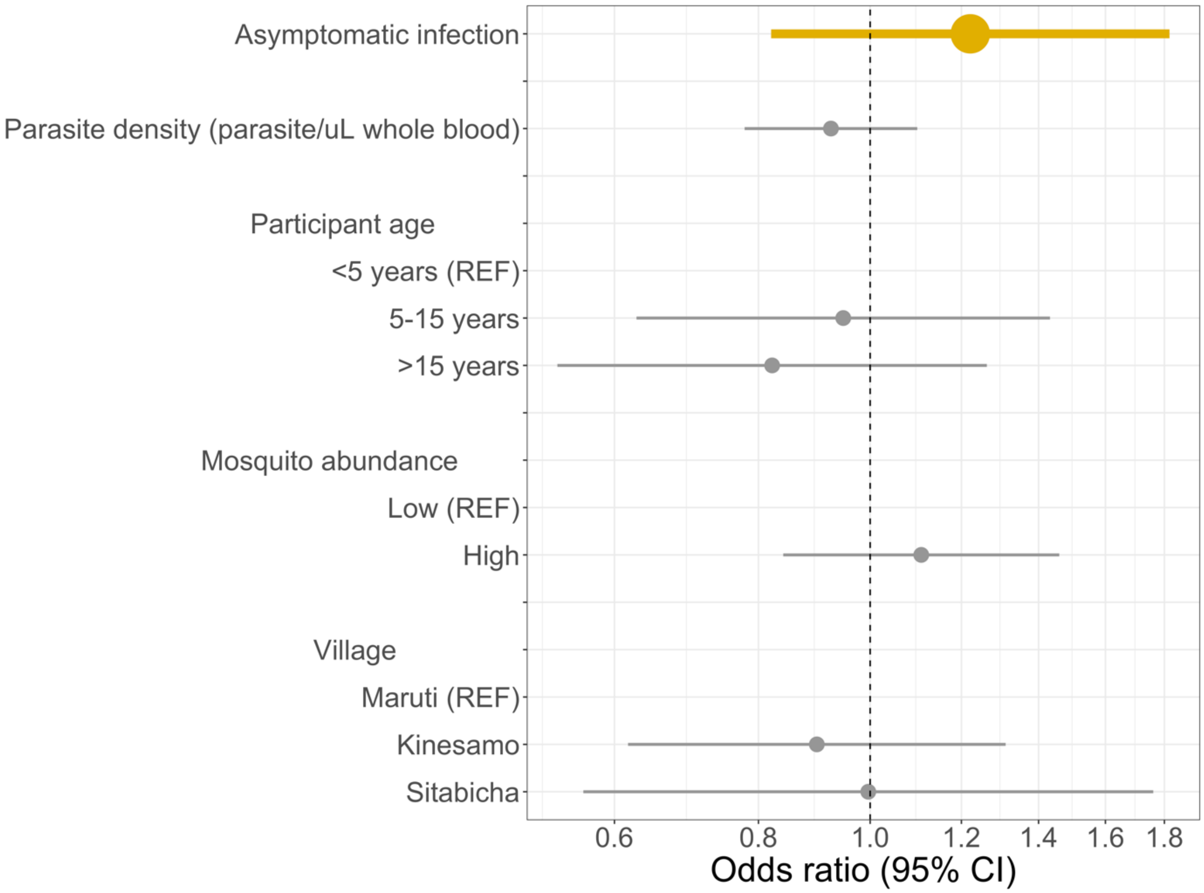

S15 Fig. Multi-level logistic regression results for odds of a participant-to-mosquito malaria transmission from participants with asymptomatic compared to symptomatic infections using the *pfama1* malaria gene target. We ran a multi-level logistic regression using the continuous coding of $P(TE_{all})$ and *pfama1* haplotypes. The model controlled for covariates: parasite density in the participant samples in parasites/μL (linear), age (categorized: <5 years, 5-15 years, >15 years), the mosquito abundance (binary: <75 mosquitoes, ≥75 mosquitoes), and village. Model results suggested higher odds of asymptomatic compared to symptomatic malaria transmission to mosquitoes (N=3160 participant-mosquito pairings), but results were not statistically significant. Data are presented as odds ratios (dots) with 95% confidence intervals (bars).

Supplementary Tables

Table S1. Comparison of participant-mosquito pairs among 65 participants included in within-participant modeling to full data set of all participants

	**Analysis data set 65 participants (1565 pairings)**	**Full data set 198 participants (3727 pairings)**	**P-value**
**Participant-level covariates**			
**Parasite density** (parasites/μL), Median (IQR)	290.55 (3654.96)	43.49 (731.76)	<0.001[a]
**Age**, N (%)			<0.001[b]
<5 years	179 (11.44)	438 (11.75)	
5-15 years	1105 (70.61)	1806 (48.46)	
>15 years	281 (17.96)	1483 (39.79)	
**Number of *pfcsp* haplotypes**, Median (IQR)	1.00 (2.00)	3.00 (6.00)	0.211[a]
**Number of infections per participant,** Median (IQR)	3.00 (2.00)	2.00 (3.00)	<0.001[a]

Abbreviations: IQR, interquartile range; *pfcsp, Plasmodium falciparum* circumsporozoite protein

[a] Two-sided Wilcoxon Rank Sum test with continuity correction and Bonferroni correction for repeated measures

[b] Two-sided Pearson's χ^2 test with Bonferroni correction for repeated measures

Table S2. Differences between participant-mosquito pairs that were excluded from the analysis due to time and distance constraints across model covariates

	Asymptomatic infections (N=132,593)	Symptomatic infections (N=22,965)
**Participant-level covariates**		
**Parasite density** (parasites/μL), Median (IQR)	6.73 (166.45)	1,545.74 (6,370.95)
**Age**, N (%)		
<5 years	12,994 (9.80)	3,033 (13.21)
5-15 years	68,061 (51.33)	14,493 (63.11)
>15 years	51,538 (38.87)	5,439 (23.68)
**Mosquito abundance,** N (%)		
Low	115,911 (87.42)	15,128 (65.87)
High	16,682 (12.58)	7,837 (34.13)
**Number of *pfcsp* haplotypes**, Median (IQR)	3.00 (5.00)	1.00 (2.00)
**Village**, N (%)		
Maruti	45,463 (34.29)	7,544 (32.85)
Kinesamo	37,124 (28.00)	6,267 (27.29)
Sitabicha	50,006 (37.71)	9,154 (39.86)
**Participant-mosquito pair-level covariates**		
**Probability of transmission**, Median (IQR)		
Across all variables[#]	0.00 (0.00)	0.00 (0.00)
Time interval	0.00 (0.00)	0.00 (0.00)
Distance interval	0.00 (0.51)	0.00 (0.47)
pfcsp haplotype sharing and prevalence*	0.09 (0.24)	0.00 (0.17)
For those that shared *pfcsp* haplotypes	0.20 (0.19)	0.20 (0.23)
**Number *pfcsp* haplotypes shared**, Median (IQR)**	1.00 (2.00)	0.00 (1.00)
For those that shared *pfcsp* haplotypes	2.00 (2.00)	1.00 (0.00)

Abbreviations: IQR, interquartile range; *pfcsp, Plasmodium falciparum* circumsporozoite protein

[#] The probability of transmission across all variables was 0.00 because the participant-mosquito pairs were not within the distance and time restraints to be a likely participant-to-mosquito transmission.

*The probability of transmission based on the *pfcsp* haplotype sharing and prevalence is shown for all pairings regardless on if they shared haplotypes or not.

**The number of *pfcsp* haplotypes shared is shown for all pairs regardless on if they shared haplotypes or not.

Table S3. Results of multi-level logistic regression models of probability of a transmission event using different functional forms of the *P. falciparum* parasite density in humans

Coding Choice / Term	Coefficient	SE	Log Likelihood	AIC
Linear			-601.0	1210.0
Parasite density	1.13	1.07		
Quadratic			-599.3	1208.7
Parasite density	0.70	1.34		
Parasite density squared	1.08	1.04		
Cubic			-599.3	1210.6
Parasite density	0.75	1.48		
Parasite density squared	1.03	1.21		
Parasite density cubed	1.01	1.02		
Binary			0598.3	1204.7
<100 p/μL (under cRDT detection)	Ref	Ref		
≥100 p/μL (over cRDT detection)	0.60	1.20		
Categorical			-596.0	1203.9
< 1.93 p/μL	Ref	Ref		
≥ 1.93 and < 51.64 p/μL	1.34	1.25		
≥ 51.64 and 773.53 p/μL	0.57	1.31		
≥ 773.53 p/μL	0.75	1.29		
Natural Log			-600.1	1208.2
Parasite density ln	0.95	1.03		

Abbreviations: SE, standard error; AIC, Akaike information criteria; cRDT, conventional rapid diagnostic test

Table S4. Results of multi-level logistic regression models of probability of a transmission event using different functional forms of participant age

Coding Choice / Term	Coefficient	SE	Log Likelihood	AIC
Linear			-602.2	1212.5
Age	0.94	1.09		
Quadratic			-602.2	1214.5
Age	0.92	1.16		
Age squared	1.01	1.08		
Cubic			-599.5	1211.1
Age	0.86	1.17		
Age squared	0.69	1.20		
Age cubed	1.14	1.06		
Categorical			-600.2	1210.5
<5 years	Ref	Ref		
5-15 years	1.59	1.37		
>15 years	1.14	1.39		
Natural Log			-602.5	1212.9
Age ln	1.01	1.10		

Abbreviations: SE, standard error; AIC, Akaike information criteria

Table S5. Results of multi-level logistic regression models of probability of a transmission event using different functional forms of the total number of female *Anopheles* mosquitoes collected within one week following participant infection

Coding Choice / Term	Coefficient	SE	Log Likelihood	AIC
Linear			-602.0	1211.9
Mosquito abundance	1.09	1.09		
Quadratic			-601.9	1213.8
Mosquito abundance	1.09	1.09		
Mosquito abundance squared	1.04	1.09		
Cubic			-600.8	1213.6
Mosquito abundance	1.30	1.16		
Mosquito abundance squared	1.08	1.10		
Mosquito abundance cubed	0.92	1.06		
Binary				
<75 mosquitoes	Ref	Ref	-601.9	1211.7
75-147 mosquitoes	1.21	1.19		
Natural Log			-602.2	1212.5
Mosquito abundance ln	1.09	1.14		

Abbreviations: SE, standard error; AIC, Akaike information criteria

Table S6. Number of samples with each unique haplotype ID

Unique haplotype ID	Number of samples with haplotype[#]
1	461
2	527
3	426
4	295
5	307
6	304
7	227
8	189
10	141
9	134
11	95
17	70
24	60
16	89
12	88
22	57
19	52
13	97
20	47
18	47
14	35
21	42
29	29
23	66
25	43
26	30
33	25
28	53
40	16
35	16
30	15
31	26
27	45
32	17
15	73

39	10
43	10
36	9
46	11
79	6
37	12
41	14
42	6
34	15
44	4
50	8
53	10
68	4
38	5
58	6
61	3
66	7
105	3
45	3
47	3
51	4
55	3
56	9
65	2
72	3
75	3
161	2
49	3
54	4
63	6
67	2
70	2
78	1
81	1
85	1
89	1
91	2
94	1

101	1
104	1
106	1
109	1
113	1
115	1
120	1
129	1
137	1
138	3
140	1
142	1
145	1
155	1
156	1
157	1
158	1
165	1
168	1
172	1
176	1
177	1
184	2
185	1
186	1
188	1
194	1
203	1
205	1
207	1
210	1
218	1
220	1
223	1
224	1
229	1
231	1
234	1

241	1
246	1
247	1
248	1
253	1
256	1
261	1
265	1
267	1
269	1
275	1
277	1
278	1
279	1
281	1
284	1
292	1
294	1
297	1
298	1
48	1
57	6
59	1
60	4
62	5
64	1
71	1
73	1
74	1
76	1
77	1
82	1
83	1
84	1
87	3
88	1
90	1
92	1

95	2
96	1
98	2
99	1
102	2
103	1
107	1
110	1
112	1
114	1
116	2
117	1
118	2
119	1
121	1
123	1
124	1
125	1
126	1
128	1
133	1
134	1
135	1
136	1
147	1
148	1
149	2
150	1
151	1
152	1
159	1
160	1
162	1
163	1
166	1
167	1
169	1
170	1

173	2
179	1
180	1
183	1
187	1
189	1
191	1
192	1
196	1
197	1
199	1
201	1
202	1
204	1
212	1
214	1
217	1
219	1
221	1
222	1
226	1
227	1
228	1
230	1
233	1
237	1
238	1
239	1
240	1
244	1
250	1
251	1
254	1
255	1
258	1
268	1
271	1
276	1

285	1
288	1
291	1
293	1

This represents the total number of samples each haplotype was identified in, including infected mosquitoes, symptomatic infected participants, and asymptomatic infected participants. The table is ordered by frequency of each unique haplotype ID.

Table S7. Round 1 primers for amplicon deep sequencing

Name	Sequence*
PfcspOH-F	TCGTCGGCAGCGTCAGATGTGTATAAGAGACAGTTAAGGAACAAGAAGGATAATACCA
PfcspOH-R	GTCTCGTGGGCTCGGAGATGTGTATAAGAGACAGAAATGACCCAAACCGAAATG
PfamaOH-F	TCGTCGGCAGCGTCAGATGTGTATAAGAGACAGTCAGGGAAATGTCCAGTATTTG
PfamaOH-R	GTCTCGTGGGCTCGGAGATGTGTATAAGAGACAGGGACCATTATTTTCTTGAGCTG

*In the above sequences, the non-underlined, non-shaded sequence is complementary to the target sequence in the corresponding *Plasmodium falciparum* circumsporozoite protein *(pfcsp)* or apical membrane antigen-1 *(pfama1)* gene. The underlined portion is the overhang sequence that is included in the PCR products for downstream paired-end Illumina MiSeq sequencing. The shaded part of the underlined sequence is targeted by the second-round primers. Primers are the same as Nelson *et al.*[3]

Table S8. Round 2 primers for amplicon deep sequencing

Name	Index	Sequence*
P5MiSeqi1	AACCAAGG	**AATGATACGGCGACCACCGAGATCTACAC**AACCAAGGTCGTCGGCAGCGTC
P5MiSeqi2	AAGGTACG	**AATGATACGGCGACCACCGAGATCTACAC**AAGGTACGTCGTCGGCAGCGTC
P5MiSeqi3	ACCTACCT	**AATGATACGGCGACCACCGAGATCTACAC**ACCTACCTTCGTCGGCAGCGTC
P5MiSeqi4	ACGTGTTG	**AATGATACGGCGACCACCGAGATCTACAC**ACGTGTTGTCGTCGGCAGCGTC
P5MiSeqi5	ACTGGACT	**AATGATACGGCGACCACCGAGATCTACAC**ACTGGACTTCGTCGGCAGCGTC
P5MiSeqi6	AGAGACTG	**AATGATACGGCGACCACCGAGATCTACAC**AGAGACTGTCGTCGGCAGCGTC
P5MiSeqi7	AGTCGACT	**AATGATACGGCGACCACCGAGATCTACAC**AGTCGACTTCGTCGGCAGCGTC
P5MiSeqi8	ATATGCCG	**AATGATACGGCGACCACCGAGATCTACAC**ATATGCCGTCGTCGGCAGCGTC
P5MiSeqi9	CAACCATG	**AATGATACGGCGACCACCGAGATCTACAC**CAACCATGTCGTCGGCAGCGTC
P5MiSeqi10	CACAGTGT	**AATGATACGGCGACCACCGAGATCTACAC**CACAGTGTTCGTCGGCAGCGTC
P5MiSeqi11	CAGAAGTG	**AATGATACGGCGACCACCGAGATCTACAC**CAGAAGTGTCGTCGGCAGCGTC
P5MiSeqi12	CAGTGACT	**AATGATACGGCGACCACCGAGATCTACAC**CAGTGACTTCGTCGGCAGCGTC
P5MiSeqi13	CATGTGGT	**AATGATACGGCGACCACCGAGATCTACAC**CATGTGGTTCGTCGGCAGCGTC
P5MiSeqi14	CTTCGAAG	**AATGATACGGCGACCACCGAGATCTACAC**CTTCGAAGTCGTCGGCAGCGTC
P5MiSeqi15	CATCGATG	**AATGATACGGCGACCACCGAGATCTACAC**CATCGATGTCGTCGGCAGCGTC
P5MiSeqi16	CGTAGGAA	**AATGATACGGCGACCACCGAGATCTACAC**CGTAGGAATCGTCGGCAGCGTC
P5MiSeqi17	CTAGTGGT	**AATGATACGGCGACCACCGAGATCTACAC**CTAGTGGTTCGTCGGCAGCGTC
P5MiSeqi18	CTCTGACT	**AATGATACGGCGACCACCGAGATCTACAC**CTCTGACTTCGTCGGCAGCGTC
P5MiSeqi19	CTGTGAGT	**AATGATACGGCGACCACCGAGATCTACAC**CTGTGAGTTCGTCGGCAGCGTC
P5MiSeqi20	GAACCTTG	**AATGATACGGCGACCACCGAGATCTACAC**GAACCTTGTCGTCGGCAGCGTC
P5MiSeqi21	GACATCTG	**AATGATACGGCGACCACCGAGATCTACAC**GACATCTGTCGTCGGCAGCGTC
P5MiSeqi22	GAGAGACT	**AATGATACGGCGACCACCGAGATCTACAC**GAGAGACTTCGTCGGCAGCGTC
P5MiSeqi23	GATCGAAG	**AATGATACGGCGACCACCGAGATCTACAC**GATCGAAGTCGTCGGCAGCGTC
P5MiSeqi24	GCAATAGG	**AATGATACGGCGACCACCGAGATCTACAC**GCAATAGGTCGTCGGCAGCGTC
P7MiSeqi25	GCTACCTT	**CAAGCAGAAGACGGCATACGAGAT**GCTACCTTGTCTCGTGGGCTCGG
P7MiSeqi26	GACACTGT	**CAAGCAGAAGACGGCATACGAGAT**GACACTGTGTCTCGTGGGCTCGG
P7MiSeqi27	GGTTCCTT	**CAAGCAGAAGACGGCATACGAGAT**GGTTCCTTGTCTCGTGGGCTCGG
P7MiSeqi28	GTCATCAG	**CAAGCAGAAGACGGCATACGAGA**TGTCATCAGGTCTCGTGGGCTCGG
P7MiSeqi29	GTGAGTCT	**CAAGCAGAAGACGGCATACGAGA**TGTGAGTCTGTCTCGTGGGCTCGG
P7MiSeqi30	GTTCGATG	**CAAGCAGAAGACGGCATACGAGA**TGTTCGATGGTCTCGTGGGCTCGG
P7MiSeqi31	TACGATCG	**CAAGCAGAAGACGGCATACGAGA**TTACGATCGGTCTCGTGGGCTCGG
P7MiSeqi32	TCACTCTG	**CAAGCAGAAGACGGCATACGAGA**TTCACTCTGGTCTCGTGGGCTCGG
P7MiSeqi33	TCTCCAGT	**CAAGCAGAAGACGGCATACGAGA**TTCTCCAGTGTCTCGTGGGCTCGG
P7MiSeqi34	TGACTCAG	**CAAGCAGAAGACGGCATACGAGA**TTGACTCAGGTCTCGTGGGCTCGG
P7MiSeqi35	TGGTTCCT	**CAAGCAGAAGACGGCATACGAGA**TTGGTTCCTGTCTCGTGGGCTCGG
P7MiSeqi36	TGTGACTG	**CAAGCAGAAGACGGCATACGAGA**TTGTGACTGGTCTCGTGGGCTCGG
P7MiSeqi37	TGTGACTG	**CAAGCAGAAGACGGCATACGAGA**TTGTGACTGGTCTCGTGGGCTCGG
P7MiSeqi38	ACACGACT	**CAAGCAGAAGACGGCATACGAGA**TACACGACTGTCTCGTGGGCTCGG
P7MiSeqi39	ACTGTCAG	**CAAGCAGAAGACGGCATACGAGA**TACTGTCAGGTCTCGTGGGCTCGG
P7MiSeqi40	AGTCTCAG	**CAAGCAGAAGACGGCATACGAGA**TAGTCTCAGGTCTCGTGGGCTCGG
P7MiSeqi41	ATCCAACG	**CAAGCAGAAGACGGCATACGAGA**TATCCAACGGTCTCGTGGGCTCGG
P7MiSeqi42	CAACCTAG	**CAAGCAGAAGACGGCATACGAGA**TCAACCTAGGTCTCGTGGGCTCGG
P7MiSeqi43	CACATCAG	**CAAGCAGAAGACGGCATACGAGA**TCACATCAGGTCTCGTGGGCTCGG
P7MiSeqi44	CAGACTGT	**CAAGCAGAAGACGGCATACGAGA**TCAGACTGTGTCTCGTGGGCTCGG
P7MiSeqi45	GTCTACAG	**CAAGCAGAAGACGGCATACGAGA**TGTCTACAGGTCTCGTGGGCTCGG
P7MiSeqi46	GTGATGAG	**CAAGCAGAAGACGGCATACGAGA**TGTGATGAGGTCTCGTGGGCTCGG
P7MiSeqi47	GTTGGTTG	**CAAGCAGAAGACGGCATACGAGA**TGTTGGTTGGTCTCGTGGGCTCGG
P7MiSeqi48	TACGTACG	**CAAGCAGAAGACGGCATACGAGA**TTACGTACGGTCTCGTGGGCTCGG

* In the above sequences, the bolded sequence is the P5 or P7 adaptor, and the shaded sequence is complementary to the overhang sequence in the round one products. The non-

shaded, non-bolded sequences are the 8-mer indices, selected from Hamady *et al.*[14] and Herbold *et al.*[15] Primers are the same as Nelson *et al.*[3]

Supplementary References

1. Plowe, C. V., Djimde, A., Bouare, M., Doumbo, O. & Wellems, T. E. Pyrimethamine and proguanil resistance-conferring mutations in Plasmodium falciparum dihydrofolate reductase: Polymerase chain reaction methods for surveillance in Africa. *Am. J. Trop. Med. Hyg.* **52**, 565–568 (1995).
2. Taylor, S. M. *et al.* Direct estimation of sensitivity of Plasmodium falciparum rapid diagnostic test for active case detection in a high-transmission community setting. *Am. J. Trop. Med. Hyg.* **101**, 1416–1423 (2019).
3. Nelson, C. S. *et al.* High-resolution micro-epidemiology of parasite spatial and temporal dynamics in a high malaria transmission setting in Kenya. *Nat. Commun.* **10**, (2019).
4. Illumina. Illumnia Miseq. https://www.illumina.com/systems/sequencing-platforms/miseq.html (2018).
5. Bushnell, B. BBMap short-read aligner, and other bioinformatics tools. (2015).
6. Bolger, A. M., Lohse, M. & Usadel, B. Trimmomatic: A flexible trimmer for Illumina sequence data. *Bioinformatics* **30**, 2114–2120 (2014).
7. Martin, M. *Embnet.news : European Molecular Biology Network newsletter. Cutadapt removes adapter sequences from high-throughput sequencing reads* vol. 17 (EMBnet, Administration Office, 2011).
8. Callahan, B. *et al.* High-resolution sample inference from Illumina amplicon data. *Nat. Methods* **13**, 581–583 (2016).
9. R Core Team. R: A language and environment for statistical computing. (2019).
10. Early, A. M. *et al.* Detection of low-density Plasmodium falciparum infections using amplicon deep sequencing. *Malar. J.* **18**, 219 (2019).
11. Aurrecoechea, C. *et al.* PlasmoDB: A functional genomic database for malaria parasites. *Nucleic Acids Res.* **37**, 539–543 (2009).

12. MalariaGEN Resource Centre. Pf3k: MalariaGen. (2020).

13. Neafsey, D. E. *et al.* Genetic Diversity and Protective Efficacy of the RTS,S/AS01 Malaria Vaccine. *N. Engl. J. Med.* **373**, 2025–2037 (2015).

14. Hamady, M., Walker, J. J., Harris, J. K., Gold, N. J. & Knight, R. Error-correcting barcoded primers allow hundreds of samples to be pyrosequenced in multiplex. *Nat. Methods* **5**, 235–237 (2008).

15. Herbold, C. W. *et al.* A flexible and economical barcoding approach for highly multiplexed amplicon sequencing of diverse target genes. *Front. Microbiol.* **6**, 1–8 (2015).
