# Supplementary material for: Genotyping cognate Plasmodium falciparum in humans and mosquitoes to estimate onward transmission of asymptomatic infections
Source: Nat Commun. 2021 Feb 10;12:909. doi: 10.1038/s41467-021-21269-2 (PMC7875998; doi:10.1038/s41467-021-21269-2)
Supplement: Supplementary file 3 — Reporting Summary [file 41467_2021_21269_MOESM3_ESM.pdf]

nature research

Corresponding author(s): Steve Taylor

Last updated by author(s): 01/08/2021

Reporting Summary

Nature Research wishes to improve the reproducibility of the work that we publish. This form provides structure for consistency and transparency in reporting. For further information on Nature Research policies, see our Editorial Policies and the Editorial Policy Checklist.

Statistics

For all statistical analyses, confirm that the following items are present in the figure legend, table legend, main text, or Methods section.

n/a	Confirmed	
☐	☒	The exact sample size (*n*) for each experimental group/condition, given as a discrete number and unit of measurement
☐	☒	A statement on whether measurements were taken from distinct samples or whether the same sample was measured repeatedly
☐	☒	The statistical test(s) used AND whether they are one- or two-sided<br>*Only common tests should be described solely by name; describe more complex techniques in the Methods section.*
☐	☒	A description of all covariates tested
☐	☒	A description of any assumptions or corrections, such as tests of normality and adjustment for multiple comparisons
☐	☒	A full description of the statistical parameters including central tendency (e.g. means) or other basic estimates (e.g. regression coefficient) AND variation (e.g. standard deviation) or associated estimates of uncertainty (e.g. confidence intervals)
☐	☒	For null hypothesis testing, the test statistic (e.g. *F*, *t*, *r*) with confidence intervals, effect sizes, degrees of freedom and *P* value noted<br>*Give P values as exact values whenever suitable.*
☒	☐	For Bayesian analysis, information on the choice of priors and Markov chain Monte Carlo settings
☒	☐	For hierarchical and complex designs, identification of the appropriate level for tests and full reporting of outcomes
☐	☒	Estimates of effect sizes (e.g. Cohen's *d*, Pearson's *r*), indicating how they were calculated

Our web collection on statistics for biologists contains articles on many of the points above.

Software and code

Policy information about availability of computer code

Data collection	Clinical and demographic data were collected using Microsoft Access
Data analysis	R code is available to access on GitHub: https://github.com/duke-malaria-collaboratory/human_mosquito_manuscript.git.

For manuscripts utilizing custom algorithms or software that are central to the research but not yet described in published literature, software must be made available to editors and reviewers. We strongly encourage code deposition in a community repository (e.g. GitHub). See the Nature Research guidelines for submitting code & software for further information.

Data

Policy information about availability of data

All manuscripts must include a data availability statement. This statement should provide the following information, where applicable:

- Accession codes, unique identifiers, or web links for publicly available datasets
- A list of figures that have associated raw data
- A description of any restrictions on data availability

Source data and code are available on GitHub: https://github.com/duke-malaria-collaboratory/human_mosquito_manuscript.git. Sequence data including raw reads and parasite haplotypes are available from NCBI (PRJNA646940).

Field-specific reporting

Life sciences study design

All studies must disclose on these points even when the disclosure is negative.

Sample size	The sample size of the prospective cohort was fixed at 36 households and their members. This number was chosen in order to enable sufficient capture of both human and mosquito P falciparum infections during the 14 month study period.
Data exclusions	We excluded participants a posteriori who had less than 2 of the expected 14 months of followup or had no malaria infections, owing to their inability to contribute to the primary analysis.
Replication	The primary units of analysis are the parasite haplotypes recovered from infected hosts and each target from each infection was amplified and then sequencing singly without replication.
Randomization	This was not a randomized study because it was an observational study of naturally-occurring malaria infections. The independent variable was an asymptomatic malaria infection, to which it is not feasible to randomize a person.
Blinding	There was no group allocation and therefore no masking.

Reporting for specific materials, systems and methods

We require information from authors about some types of materials, experimental systems and methods used in many studies. Here, indicate whether each material, system or method listed is relevant to your study. If you are not sure if a list item applies to your research, read the appropriate section before selecting a response.

Materials & experimental systems

n/a	Involved in the study
☒	☐ Antibodies
☒	☐ Eukaryotic cell lines
☒	☐ Palaeontology and archaeology
☐	☒ Animals and other organisms
☐	☒ Human research participants
☒	☐ Clinical data
☒	☐ Dual use research of concern

Methods

n/a	Involved in the study
☒	☐ ChIP-seq
☒	☐ Flow cytometry
☒	☐ MRI-based neuroimaging

Animals and other organisms

Policy information about studies involving animals; ARRIVE guidelines recommended for reporting animal research

Laboratory animals	None
Wild animals	None
Field-collected samples	Anopheline mosquitos were collected as described in the manuscript from enrolled households using a vacuum aspirator, morphologically assessed following chloroform treatment, dissected head from thorax/abdomen using sterilized forceps, and these were each placed into individual labeled microfuge tubes.
Ethics oversight	Moi University, Duke University, UNC

Note that full information on the approval of the study protocol must also be provided in the manuscript.

Human research participants

Policy information about studies involving human research participants

Population characteristics	Children (> 1y) and adults resident in the enrolled 36 households were enrolled after informed consent (and assent if applicable). All members of randomly-selected households were offered enrollment irrespective of sex and other demographic or clinical characteristics, the only requirement other than the age criteria being that they self-reported sleeping in the house regularly. All were residents of villages in Bungoma County, Kenya.
Recruitment	Households were invited to participate if present in 1 of 3 villages selected on the basis of varying malaria transmission intensity in a prior cross-sectional study. To do so, we enrolled an index household in each village at random and then neighboring households radiating outwards until 12 households were enrolled per village in a natural grouping. Two households were replaced with neighboring households when the entire household migrated.
Ethics oversight	The study was approved by the Institutional Research Ethics Committee of Moi University (2017/36) and the Internal Review Boards of Duke University (Pro00082000) and the University of North Carolina at Chapel Hill (19-1273).

Note that full information on the approval of the study protocol must also be provided in the manuscript.
